# Sepsis and Oxidative Stress in the Newborn: From Pathogenesis to Novel Therapeutic Targets

**DOI:** 10.1155/2018/9390140

**Published:** 2018-08-02

**Authors:** Chiara Poggi, Carlo Dani

**Affiliations:** ^1^Division of Neonatology and Neonatal Intensive Care, Department of Mother and Child Care, Careggi University Hospital, Florence, Italy; ^2^Department of Neurosciences, Psychology, Drug Research, and Child Health, University of Florence, Florence, Italy

## Abstract

Sepsis is at present one of the leading causes of morbidity and mortality in the neonatal population. Together with inflammation, oxidative stress is involved in detrimental pathways activated during neonatal sepsis, eventually leading to organ dysfunction and death. The redox cascade during sepsis is mainly initiated by IL-6 and IL-8 stimulation in newborns and includes multiple noxious processes, as direct cell damage induced by reactive oxygen species, activation of gene expression leading to amplification of inflammation and oxidative stress, and impairment of mitochondrial function. Once proinflammatory and prooxidant pathways are established as stimulated by causing pathogens, self-maintaining unfavorable redox cycles ensue, leading to oxidative stress-related cellular damage, independently from the activating pathogens themselves. Despite antioxidant systems are induced during neonatal sepsis, as an adaptive response to an increased oxidative burden, a condition of redox imbalance favoring oxidative pathways occurs, resulting in increased markers of oxidative stress damage. Therefore, antioxidant treatment would exert beneficial effects during neonatal sepsis, potentially interrupting prooxidant pathways and preventing the maintenance of detrimental redox cycles that cannot be directly affected by antibiotic treatment. Among others, antioxidant agents investigated in clinical settings as adjunct treatment for neonatal sepsis include melatonin and pentoxifylline, both showing promising results, while novel antioxidant molecules, as edaravone and endothelin receptor antagonists, are at present under investigation in animal models. Finally, mitochondria-targeted antioxidant treatments could represent an interesting line of research in the treatment of neonatal sepsis.

## 1. Introduction

Despite general improvement in intensive care of acutely ill newborns, sepsis is still among the leading causes of death in the neonatal population worldwide [1]. On a whole, neonatal sepsis was reported to occur in 1 every 1000 live births [2]; however, incidence as high as 3% to 20% were reported in the population of preterm newborns, due to the presence of multiple coexisting risk factors for nosocomial sepsis [3]. Mortality due to neonatal sepsis is strictly dependent on the causative pathogen and on the gestational age of the patients, with a mortality rate as high as 20% observed in very preterm newborns [2, 3].

According to the guidelines of the International Pediatric Sepsis Consensus Conference, neonatal sepsis is defined as a clinical syndrome characterized by the presence of both infection and systemic inflammatory response syndrome (SIRS) [4, 5]. SIRS includes inadequate core temperature stability, tachycardia or bradycardia, tachypnea or unexplained need for mechanical ventilation, and leukocyte count elevated or depressed for postnatal age [4]. It is at present widely accepted that the infective insult due to the invasion of sterile tissues by pathogens merely represents the initiation of sepsis, while the process leading to the sepsis syndrome is subsequently maintained by a cascade of inflammatory and oxidative mechanisms that, once activated, act independently from the presence of the pathogens themselves [6].

It was demonstrated that, at least in adults, the immune system shows a typical two-phase response during sepsis, characterized by an initial increase of proinflammatory mediators followed by a shift towards anti-inflammatory cytokines, anergy of T-cells, and also apoptosis-induced loss of cells of the adaptive immune system in the most severe cases [7]. Activation of the immune system during sepsis is paralleled by a complex chain of redox events in both adults [8] and newborns [9], which partially differ among the two populations. The redox cascade initiated by immune activation includes generation of consistent amount of reactive oxygen species (ROS) and reactive nitrogen species (RNS), activation of DNA transcription processes, and mitochondrial functional impairment, eventually leading to multiple organ dysfunction and death [8, 9].

## 2. Redox Status in Neonatal Sepsis

### 2.1. The Redox Sepsis Cascade

While tumor necrosis factor-alpha (TNF-alpha) plays a pivotal role in the onset of adult sepsis [8], interleukin- (IL-) 6 and IL-8 represent the cytokines mainly involved in the initiation of the sepsis cascade in the newborn [9]. Levels of IL-6 and IL-8 are significantly increased in septic newborns in comparison to healthy controls, in both early-onset (EOS) and late-onset sepsis (LOS) [10, 11] at least for the first 12–24 hours from the onset of sepsis [10], and were proposed as useful markers for the early diagnosis of sepsis in newborns in experimental settings, and, when available, also in clinical practice [11, 12]. However, recent evidences suggest that the cytokines expression profile may consistently differ among septic newborns, according to the timing of sepsis presentation and to the patients' gestational age. In a small cohort of term and late preterm newborns, TNF-alpha increased in both EOS and LOS in comparison to healthy controls, but only patients with LOS showed also increased levels of IL-6 and IL-10 [13]. Moreover, while proinflammatory cytokines as TNF-alpha and IL-6 were upregulated in the acute phase of sepsis, anti-inflammatory cytokines as IL-4 and IL-10 were preferentially overexpressed during the subacute phase [13], providing evidence that the two-phase immune response, typical of adult sepsis, would likely occur also in newborns. The comparison of immune response during sepsis in preterm newborn ≥ 32 or <32 weeks of gestational age showed that mediators of innate immune response, as C-reactive protein (CRP) and SC5b-9, are increased in both groups, but proinflammatory cytokines as interferon-gamma, TNF-alpha, and IL-6 are upregulated only in the subgroup with gestational age ≥ 32 weeks, while both groups showed increased levels of anti-inflammatory cytokines, as IL-4 and IL-10 [14]. The differential profile of cytokines expression during sepsis suggests that partially different pathways could be involved in the initial trigger of the sepsis process at different gestational ages.

It has been proposed that, following the release of proinflammatory cytokines, several oxidative stress-related pathways are activated through different mechanisms, triggering the initiation of a self-maintaining “sepsis redox cycle,” finally leading to cell oxidative damage and mitochondria impairment [9]. According to this model, the cytokine-induced activation of nuclear factor kappa-light-chain-enhancer of activated B cells (NF-*κ*B) constitutes the first step of the process [9]. Several observations are consistent with NF-*κ*B activation during neonatal sepsis [15, 16], and in a mouse model of *group B Streptococcus* neonatal sepsis, the pathway mediated by c-Jun N-terminal kinase was demonstrated to play a pivotal role in the orchestration of inflammation during sepsis, as its inhibition significantly suppressed proinflammatory cytokines production [17]. Similarly, it was recently demonstrated that protein kinase D is essential for NF-*κ*B pathway activation during *group B Streptococcus* sepsis [18]. NF-*κ*B acts as a powerful transcription factor which binds to DNA and activates the transcription of several different genes including inducible nitric oxide synthase (iNOS) and cyclooxygenase 2 (COX-2) [8, 9], leading to increased production of nitric oxide (NO) and superoxide, respectively [9]. Superoxide levels are further increased by the cytokine-induced direct activation of NADPH oxidase [9]. In a cardiomyocyte model of neonatal sepsis, challenge with Gram-negative lipopolysaccharide (LPS) induced NADPH overexpression, leading to COX-2 overexpression through a MAP-kinase/NF-*κ*B-dependent mechanism [19]. Therefore, both direct activity of NADPH oxidase and NADPH-induced COX-2 upregulation can contribute to increase cytoplasmic superoxide [6]. Superoxide is then dismutated to H_2_O_2_ by cytoplasmic CuZn-superoxide dismutase (SOD) [6]. In a neonatal mouse model of sepsis-induced lung injury, LPS-induced lung cytokines expression, neutrophils influx, and NF-*κ*B translocation were suppressed in NADPH oxidase-deficient animals [20]. Consistent with these observations, also LPS-induced matrix metalloproteinase expression was reduced, as well as alveolar adverse remodeling characterized by reduced number of alveoli and complexity of lung alveolarization [20]. These observations suggest that NADPH oxidase may play a key role in determining the disruption of lung architecture, typical of bronchopulmonary dysplasia (BPD) [21] in septic preterm newborns.

Increased levels of NO were demonstrated in neonatal sepsis [22, 23]. Particularly, preterm newborns < 27 weeks of gestational age presented lower basal levels of NO in comparison to more mature patients, but produced larger amounts of NO during the first 2 days of bacteremia, suggesting that both the basal production of NO and the modulation of NO production may be related to gestational age [23]. In a cohort of term or near term newborn with EOS, circulating NO was significantly higher in comparison to controls [24]. Moreover, iNOS-deficient mice model presented lower degree of inflammation following exposure to *Escherichia coli* [25], and iNOs overexpression was also demonstrated by real-time PCR in neonatal respiratory epithelial cells challenged with *Staphylococcus aureus* and *Staphylococcus epidermidis*, together with proinflammatory cytokines upregulation [26].

NO, along with ROS, directly inhibits electron transport chain in the mitochondria [27, 28], resulting in impaired energy production and accumulation of mitochondrial superoxide [9, 27, 28]. Mitochondrial dysfunction was demonstrated to be the central core of deleterious proinflammatory and prooxidant routes in adult sepsis [8]. Within the mitochondrion, superoxide can react with NO to produce peroxinitrite, which in turn decomposes to hydroxyl radical and nitrogen dioxide [9]. These highly reactive species further affect mitochondrial functionality [9], and in cellular model of neonatal sepsis, peroxinitrite was shown to suppress mitochondrial function [29], thus favoring the maintenance of a detrimental pathway within the mitochondrion itself. Alternatively, superoxide anion derived by dysfunctional electron transport chain (ETC) can undergo dismutation to H_2_O_2_ by MnSOD within the mitochondrion, which is then released in the cytosol [9]. Therefore, as a result of NADPH oxidase activity, COX-2 overexpression, and dysfunctional mitochondrial ETC, increasing amounts of H_2_O_2_ accumulate in the cytosol and activate NF-*κ*B, thus completing a detrimental self-maintaining redox loop [9].

In preterm newborns, oxidative pathway activation during sepsis would interact with a preexisting prooxidant state. Increased ROS production was demonstrated in preterm newborns, resulting from hyperoxic events and mechanical ventilation [30–32], and prematurity is well known to be associated with increased risk of oxidative stress-related diseases, as BPD and retinopathy of prematurity [31]. On the other hand, impaired antioxidant capacities were demonstrated in preterm newborns, both because of inappropriate interruption of placental-fetal transfer of antioxidant molecules and insufficient endogenous production [32]. In fact, fetal levels of antioxidant enzymes (AOEs) as SOD, catalase (CAT), glutathione peroxidase (GPx), and glutathione reductase (GR) progressively increase during gestation, paralleling the maturation of surfactant system and preterm animal models fail to induce SOD and GPx in response to oxidative challenges [33, 34], in contrast to term newborns, who were demonstrated to induce AOEs in case of fetal distress [35] or resuscitation with high FiO_2_ [36]. The imbalance of the redox status favoring prooxidative pathways in preterm newborns during sepsis could also be implicated in the pathogenesis of BPD and of the long-term neurodevelopmental sequelae observed in this population following a septic event [37]. Despite the exact pathogenetic mechanisms leading to long-term adverse outcomes in septic newborns remain to be elucidated, it has been proposed that both direct increase in oxidative burden and activation of apoptosis would play a major role in tissue impairment [8]. During ongoing development of the central nervous system, a fall in ATP levels and an increase of prooxidant species, resulting from deleterious redox cycles, would critically affect the functionality and permeability of the mitochondria [38]. Particularly, the release of cytochrome c from the mitochondria to the cytosol would activate apoptosis through caspase-mediated signaling pathways [39]. Noticeably, caspases, elements of the proapoptotic Bcl-2 family, and the apoptotic protease activating factor 1 were demonstrated to be overexpressed in neonatal brain tissue in comparison to adult brain tissue [39], indicating preferential activation of proapoptotic pathways in newborns following a noxious stimulus. Oligodendrocyte progenitors and subplate neurons, which are the cell species predominantly involved in the pathogenesis of white matter injury and periventricular leukomalacia in newborns, were proved particularly vulnerable to oxidative stress [40, 41]. Apoptosis of such cell species would also be implicated in the occurrence of visual impairment, as it would detrimentally affect the development of the optic nerve and of the visual cortex [40, 42]. Analogously, oxidative damage and apoptosis have been proposed to be involved in the pathogenesis of long-term respiratory morbidities, as BPD, since increased oxidative burden and exaggerated aspects of apoptosis were demonstrated in newborns with respiratory diseases [43–45]. In lung tissue, activation of apoptosis would result in arrest of the normal process of alveolarization, which is the typical lesion of new BPD [21].

Despite mitochondrial impairment has not been specifically investigated in the newborn, such mechanism could be relevant in the setting of neonatal sepsis. In animal models, neonatal endotoxemia was associated with impairment of carnitine palmitoyltransferase I, the enzyme that controls the entry of fatty acids into the mitochondrion and the rate of fatty acid oxidation, in the developing heart and kidney [29]. The activity of the enzyme measured in mitochondria isolated from the heart, but not from the kidney, of septic newborn rats was significantly impaired, likely because of deleterious effects of ROS on ETC [29]. Moreover, treatment with glutamine, an antioxidant agent that increases Krebs cycle intermediates and supports oxidative phosphorylation, was proved effective in reducing the circulating levels of TNF-alpha and IL-10 in newborn rats exposed to LPS, although it exerted no effect on lipid peroxidation markers and NO production [46]. These results are consistent with the previous observation of restored mitochondrial ultrastructure following exposure to glutamine treatment in a cellular model of neonatal sepsis [47]. The aspect of mitochondrial dysfunction during sepsis could be of major importance in the population of preterm newborns, as they exhibit differential basal mitochondrial functionality in comparison to term newborns [48]. Particularly, the activity of complex III and IV of respiratory chain, pyruvate dehydrogenase and citrate synthase measured on muscle mitochondria obtained from autopsy, was proved to be markedly lower in preterm newborns in comparison to older children, suggesting an age-dependent functionality of mitochondrial respiratory chain [48], which would expose more preterm newborns to an increased risk of energy deficit, and thus organ failure, particularly in case of superimposed mitochondrial impairment during sepsis.

### 2.2. Markers of Oxidative Stress and Antioxidant Defense

The profile of circulating markers of oxidative stress and of enzymatic and nonenzymatic antioxidant defenses during neonatal sepsis has been less extensively studied in comparison to adult patients (Table 1). In septic newborns, circulating levels of TNF-alpha and malondialdehyde (MDA), a common marker of polyunsaturated fatty acid peroxidation, were shown to be significantly increased in comparison to healthy controls [49, 50] along with antioxidant enzymes xanthine-oxidase, SOD, and GPx, while peroxidase and uric acid levels were suppressed [49]. Moreover, GPx in the subset of newborns with septic shock was significantly higher than in patients with sepsis but no septic shock [49], suggesting a preferential hyperactivation of antioxidant pathways related to glutathione during septic shock. In 44 newborns with sepsis, MDA, SOD, GPx, and CAT were significantly increased in comparison to controls, while uric acid and albumin levels were significantly reduced [51], and similar changes were also observed in those newborns who died because of sepsis in comparison to survivors [51].

These data are consistent with the activation of prooxidant pathways and ROS overproduction during sepsis, paralleled by an increased activity of antioxidant defense systems, which, however, cannot cope with increased oxidative burden, resulting in detrimental cellular effects, as demonstrated by increased markers of oxidative damage [49]. In accordance with these observations, in a neonatal sepsis model obtained by cecal ligation and perforation (CLP) in piglets, total hydroperoxide (TH) and biological antioxidant potentials (BAP) were both increased 1 hour after the procedure in comparison to sham animals, and BAP remained significantly higher during the 6-hour study period [52]. The increase of TH and BAP was paralleled by a significant increase of TNF-alpha and IL-6 in septic animals in comparison to controls, and a positive correlation was observed at 1-hour post-CLP between TH and BAP, TH and TNF-alpha, and BAPs and IL-6 [52], suggesting mutual interactions between inflammatory pathways and oxidative stress during neonatal sepsis. Consistent with these data, more recently, excessive ROS production was directly demonstrated by DCF fluorescence technique in vital section of renal cortex of newborn rats exposed to LPS [53].

In a cohort of 120 preterm newborns with mean gestational age of 31 weeks, including 20 patients with proven EOS, 20 patients with highly probable EOS, and 80 uninfected controls, oxidative stress and inflammatory markers in cord blood samples were proved to be significantly higher in septic patients versus controls [54]. Particularly, both protein carbonyls, a marker of protein oxidation, and thiobarbituric acid reactive species (TBARS), a marker of lipid peroxidation, were increased along with IL-6 and IL-10 levels in patients with sepsis, both proven or clinically highly probable, in comparison to controls, and in patients with proven sepsis in comparison to controls [54]. Only TBARS and IL-6, but not the other markers, were significantly increased in the group of proven sepsis versus highly probable sepsis [54]. According to ROC curve analysis, TBARS and IL-6 showed the best performance for the diagnosis of EOS among the studied markers with an area under the curve of 0.88. Multivariate logistic analysis comparing TBARS and IL-6 showed that TBARS is a better predictor of EOS and TBARS was the only marker independently associated with EOS [54]. TBARS and IL-6 levels in preterm newborns also showed a mild to moderate correlation with clinical sepsis severity score, although no correlation was demonstrated between these markers and sepsis-related mortality [55]. As some markers of oxidative stress, along with indicators of antioxidant defense, are available as point-of-care test (POCT), the confirmation of a relationship between oxidative stress markers and sepsis severity would be of major relevance in critical care settings. Particularly, available or under development POCTs for oxidative stress and antioxidant status include free oxygen radicals test [56, 57], free oxygen radicals defense test [56, 57], 8-hydroxy-2′-deoxyguanosine [58], 3-nitrotyrosine [59], CuZn SOD [60], BAP, measured as capacity of reduction of ferric to ferrous ions [57], and iridium-reducing assay, particularly sensitive to GSH [61]. Despite at present these POCTs have been studied in the adult population, they would offer several advantages for critically ill newborns, as timely manner stratification of sepsis severity and identification of a patient who would particularly benefit from antioxidant strategies, the need for small blood samples, and the possibility to monitor the response to antioxidant treatment. At present, no relationship has been established between oxidative stress markers and the development of long-term adverse outcomes in septic newborns; however, some biomarkers of inflammation, as S100B, adrenomedullin, and neuron-specific enolase, were proved to be also markers of neonatal brain damage [62]. Moreover, cord blood levels of oxidative stress markers were related to free radical-related diseases, including IVH, in preterm newborns [31], indicating that studies are needed in order to assess whether early markers could predict long-term outcome in septic newborns.

In a small cohort of septic term newborns, pretreatment levels of lipid peroxidation-derived aldehydes, MDA and 4-hydroxylalkenals (4-HDA), were demonstrated to be roughly 2-folds higher than in healthy controls [63]. In 52 newborns with LOS and mean gestational age of 35-36 weeks, plasma lipid peroxidation markers and protein carbonyls were proved to increase significantly in patients with proven or clinical sepsis in comparison to uninfected controls [64]. The study of erythrocyte selenoenzymes showed increased GPx levels in patients with clinical sepsis and reduced thioredoxin reductase levels in patients with proven sepsis in comparison to controls [64], demonstrating differential regulation of antioxidant selenoenzymes during sepsis. SOD and CAT were increased in septic patients, demonstrating an adaptive antioxidant response to oxidative stress during sepsis, while erythrocyte selenium, erythrocyte glutathione, and selenoprotein P, the main plasma selenoprotein, were markedly decreased in patients with proven or clinical sepsis in comparison to controls, suggesting consumption of selenium-containing antioxidant molecules [64].

According to the available evidence, in neonatal sepsis, both oxidative stress-related pathways and antioxidant defenses appear induced; however, redox unbalance favoring oxidative stress likely occurs, as markers of oxidative damage are increased in comparison to controls [49, 50]. Concordant with this observation, in 70 newborns with mean gestational age of 36 weeks, total oxidant state (TOS) and total antioxidant state (TAS) were both increased in septic patients in the pretreatment period versus controls and oxidative stress index (OSI), and the percentage ratio of TOS/TAS, was also increased [65], confirming the prevalence of oxidative stress pathways on antioxidant defense. TOS and TAS were also studied to monitor treatment and significantly decreased after treatment in septic patients in comparison to pretreatment levels. Moreover, paraoxonase-1 (PON-1), an enzyme located in HDL inhibiting lipoprotein oxidation in LDL [66], which is reduced in adult sepsis [67], appeared significantly lower in septic newborns before treatment in comparison to controls and also to posttreatment levels [65]. In septic patients after treatment, higher TAS levels were observed in comparison to controls, while TOS and PON-1 did not significantly differ, suggesting that compensatory antioxidant defense might continue beyond the initial oxidative burst. In contrast with these findings, in a cohort of preterm newborns with mean gestational age of 34 weeks, no differences in erythrocyte GPx, GR, and CAT were detected between septic patients and controls during the clinical course of sepsis, although in septic patients at 60 days, CAT activity was significantly increased in comparison to controls and GPx activity depressed in comparison to day 0 [68].

Chorioamnionitis is a well-known risk factor for fetal and neonatal infection, especially in preterm newborns, as about 5–17% of preterm newborns whose mother has chorioamnionitis develop EOS [69, 70]. In preterm newborns with gestational age < 30 weeks, oxidative stress markers, as isoprostanes, nonprotein-bound iron, and advanced oxidative protein products were proved to be significantly increased in cord blood of newborns of mothers with histological chorioamnionitis in comparison to the control group [71]. Moreover, a significant positive correlation was found in multivariate analysis adjusted for the main neonatal and perinatal variables between histological chorioamnionitis and cord levels of oxidative stress markers, indicating increased fetal oxidative burden during intra-amniotic infection [71]. Moreover, increased levels of oxidative markers as prolidase, matrix metalloproteinases, TOS, and OSI were demonstrated in vaginal washing fluid of healthy pregnant women with preterm premature rupture of membranes (PPROM) in comparison to controls with intact membranes, while antioxidant parameters, as PON-1 and total antioxidant capacity (TAC), were significantly lower [72]. Moreover, prolidase, matrix metalloproteinases, and oxidative-antioxidant status parameters significantly differed in women with chorioamnionitis in comparison to those without chorioamnionitis in the PPROM group and levels of prolidase, matrix metalloproteinase-13, TOS, TAC, and PON-1 were proved to predict chorioamnionitis in the PPROM group [72]. These results are partially discordant with the observation of measurable amount of oxidative stress markers in amniotic fluid in 183 pregnant women with PPROM but the absence of influence of intra-amniotic infection or histological chorioamnionitis on the levels of oxidative stress and antioxidant biomarkers [73]. Concordantly, intra-amniotic infection, histological chorioamnionitis, and funisitis did not significantly affect cord blood TAC, ferric reducing antioxidant power, TBARS, advanced glycation end products, and markers of oxidative stress in the offspring of 165 pregnancies complicated by PPROM [74]. On a whole, despite increased fetal and oxidative burden could occur as a result of PPROM and chorioamnionitis, although mixed results were reported, at present, no conclusions can be derived regarding its role in neonatal sepsis, as no data on oxidative balance are available for the subset of newborns who develop EOS as a consequence of maternal chorioamnionitis.

### 2.3. Intestinal Microbiota and Oxidative Stress

The gastrointestinal system was recently demonstrated to take part in the oxidative burst during sepsis in preterm newborns, providing evidence that host-microbiota interactions could be of major importance under septic conditions [75]. Fecal samples of 5 pairs of twins with mean gestational age of 30 weeks, each pair including one septic and one control twin, were used for microbiota analysis and genome-wide expression analysis on exfoliated intestinal cells. Induction of several genes involved in proinflammatory and prooxidant pathways was demonstrated in intestinal cells of septic newborns in comparison to controls, and such genome expression changes were paralleled by microbiota shift towards predominance of *Enterobacteria* with reduction of *Bacteroides* and *Bifidobacteria*, likely resulting from oxidative stress and low-grade inflammation in the gut mucosa [75]. A significant inverse correlation was observed between *Bacteroides* and *Bifidobacteria* and 8 genes involved in oxidative stress, and also further genes involved in TNF-alpha and IL-1beta signaling pathways [75]. These results were in agreement with a previous study of blood genome-wide expression profile in very low birthweight (VLBW) infants, demonstrating overexpression of genes involved in innate immunity and inflammation in septic patients in comparison to controls [76]. These aspects could be of major importance in the population of preterm newborns, who exhibited basal overexpression of genes related to inflammation in the gut in comparison to term newborns, as demonstrated by whole genome sequencing of stool-derived mRNA [77], and overexpressed in septic condition pathways related to IL-1 receptor kinase 2, fibroblast growth factor receptors, gap junctions, and cell division regulators [78]. Moreover, in a preterm pig model of necrotizing enterocolitis (NEC), the study of intestinal proteomics demonstrated that antibiotic treatment induced several beneficial mucosal pathways, including antioxidant ones, as CAT activity was significantly increased in comparison to untreated animals, suggesting that antibiotic treatment during NEC is associated to a more favorable redox profile, shifting towards antioxidant prevalence [79].

Intestinal microbiota has recently become a central component of the sepsis process in preterm newborns as the disruption of physiological intestinal colonization induced by aggressive antibiotic treatment was proved to favor the development of pathogen species and to be associated with adverse outcomes [80, 81]. Early empirical antibiotics administered for more than 5 days, without evidence of positive blood culture, was positively associated to increased risk of NEC and death in a large cohort of ELBW infants [82] and to increased incidence of LOS and of the combined outcome LOS-NEC-death in 365 VLBW infants [83]. It was hypothesized that high levels of circulating proinflammatory and prooxidant mediators observed in septic newborns, through an inflammatory organ cross-talk, may affect gut mucosa gene expression profile, leading to local environment inflammation and oxidative stress that, in turn, would affect microbial colonization, favoring pathogen species [75].

As antibiotics are one of the milestones of medical treatment of NEC but inappropriate antibiotic courses are related to adverse outcomes, the optimal antibiotic regimen for patients with NEC is of crucial importance in clinical settings [84, 85]. Two recent surveys reported high variability among different centers and within single centers in antibiotic treatments for NEC in terms of type and number of antibiotics and duration of treatment [84, 85]. Despite the most frequently reported regimen was the association of amoxicillin or ampicillin, gentamycin, and metronidazole [84, 85] basing on old data [86], the criteria to broaden antibiotic spectrum were variable among practitioners and for surgical patients, the duration of postsurgery antibiotic course was not standardized [85]. Two meta-analyses found insufficient evidence to make specific recommendations on the most appropriate type and duration of antibiotic treatment [87, 88]; therefore, specifically designed studies should be performed to address the optimal antibiotic regimen for patients with NEC in terms of harm/benefit ratio.

Finally, different antioxidant treatments were suggested to be effective in the prevention of NEC development in preterm newborns. Particularly, oral supplementation with lactoferrin was proved to reduce NEC occurrence in a meta-analysis [89, 90], although this evidence had low-to-moderate quality [91] and human recombinant lactoferrin was administered only in one study, while bovine lactoferrin was used in all the others [91]. The beneficial effect of lactoferrin in preserving the gut mucosa integrity is likely related to the position of lactoferrin itself on the mucosal surface, where it contrasts microbial invasion and translocation across the intestinal wall [91]. On the other hand, pentoxifylline administration showed mixed result, and in meta-analysis, did not affect NEC occurrence [92]. However, because of the low quality of evidence, specific trials investigating pentoxifylline treatment for NEC prevention and treatment are advocated [92]. N-Acetylcysteine administration in a rat model of NEC reduced gut oxidative stress damage measured as MDA, gut abnormalities, and intestinal levels of TNF-alpha, while was proved to increase local activity of antioxidant enzymes [93]. Finally, also melatonin was hypothesized to confer protection against NEC development, due to its pleiotropic and multiorgan antioxidant activities [94]; however, its potential usefulness in NEC prevention remains at present to be assessed by clinical trials.

## 3. Antioxidant Strategies in Neonatal Sepsis

Basing on evidence of increased oxidative burden in neonatal sepsis, therapeutic strategies targeting proinflammatory and prooxidant pathways would be expected to be beneficial; however, despite promising results in cellular and animal models, evidence from clinical trials is still limited. In the neonatal populations, antioxidant treatments investigated during sepsis include both direct antioxidant administration and pharmacologic inhibition of prooxidant pathways.

Melatonin demonstrated pleiotropic antiapoptotic, antioxidant, and anti-inflammatory effects in vitro and in vivo, as direct scavenging activity against ROS and other oxidizing agents and stimulation of antioxidant enzymes, as CAT, SOD, GPx, GR, and gamma-glutamylcysteine synthase, the rate-limiting enzyme in glutathione synthesis [95]. Interestingly, melatonin accumulates within the mitochondria [95, 96]; therefore, it would possibly target the local excessive ROS production, which is typical of dysfunctional mitochondria during sepsis [8, 27]. In preterm newborns, melatonin was demonstrated effective in reducing oxidative stress markers and inflammatory mediators in RDS and perinatal asphyxia [97, 98]. In a small cohort of septic term newborns, oral melatonin treatment within the first 12 hours from diagnosis (2 doses, 10 mg/kg each, administered at 1-hour interval) significantly reduced lipid peroxidation markers, MDA+4-HDA, at 1 and 4 hours after treatment [63] (Table 2). Melatonin-treated infants also showed a significant reduction in white blood cell count, absolute neutrophil count, and CRP 24 hours after treatment, while at the same time untreated septic infants presented stable white blood cell count and neutrophil count and increased CRP levels. Moreover, while 3 of 10 septic untreated infants died, no cases of death were observed in the melatonin-treated group [63]. These encouraging results are consistent with a favorable suppression of prooxidant and proinflammatory pathways induced by melatonin treatment in neonatal sepsis, occurring as early as 1 hour after oral administration [63]. Concordantly, in a recent nonrandomized trial including 40 septic newborns treated with antibiotics and melatonin (20 mg/kg, single dose) or antibiotics alone, melatonin treatment was associated with a significantly stronger reduction of CRP levels and improvement of clinical parameters in comparison to antibiotic treatment alone [99] and it was also shown to improve clinical sepsis score in comparison to antibiotic treatment alone in a cohort of 50 septic newborns [100]. These data are also concordant with the observation of beneficial effect of melatonin in newborns in the postsurgery period, showing reduced nitrite-nitrate and proinflammatory cytokines levels following melatonin administration in comparison to untreated surgical newborns [101]. Interestingly, higher melatonin levels were demonstrated in septic newborns with LOS in comparison to uninfected controls [102], suggesting that melatonin endogenous production might be upregulated during sepsis, taking part in antioxidant defense. Basing on these favorable preliminary data, randomized control trials are warranted to assess efficacy and safety of melatonin as an adjunct treatment in neonatal sepsis [103].

Promising results in antioxidant treatment for neonatal sepsis were obtained with the administration of pentoxifylline, which exerts several antioxidant and anti-inflammatory activities, as reduced glutathione level restoration, mitochondrial viability maintenance, inhibition of TNF-alpha production, preservation of proper endothelial function and of proper coagulation activity, and prevention of gastrointestinal vasoconstriction [104]. In a randomized controlled trial including 120 newborns with LOS and mean gestational age of 30 weeks, pentoxifylline administration (5 mg/kg/h i.v. for 6 hours for 6 days) was associated with reduced TNF-alpha and CRP levels, reduced need for vasopressor, shorter duration of respiratory support and antibiotic treatment, shorter hospital stay, lower incidence of disseminated intravascular coagulopathy, metabolic acidosis, and thrombocytopenia [105] (Table 2). However, no differences were observed in mortality and short-term morbidity between pentoxifylline-treated and untreated septic newborns [105, 106]. In a meta-analysis including 6 small studies, pentoxifylline administration in septic newborns was proved effective in reducing all-cause mortality and the length of hospital stay, and subgroup analysis demonstrated significantly reduced mortality in preterm infants, in infants with proven sepsis, and in infants with Gram-negative sepsis [92], leading to the conclusion that pentoxifylline may represent a beneficial adjunct treatment in neonatal sepsis, although larger trials are needed in order to define pentoxifylline efficacy and the safety profile. Interestingly, in vitro pentoxifylline was recently proved to exert more powerful anti-inflammatory effects in newborns than in adults [107]. In cord blood and adult blood stimulated with LPS, pentoxifylline treatment suppressed TLR-mediated cytokines levels, as TNF-alpha and IL-1beta, in a dose-dependent manner and this effect was more pronounced in cord blood in comparison to adult blood [107], suggesting that pentoxifylline adjunct treatment could be particularly beneficial in the neonatal population.

Lactoferrin, a normal component of human milk, is an anti-infective and antioxidant agent, acting through iron sequestration and direct detrimental effect on pathogen cell membranes [108]. In a randomized controlled trial including 472 VLBW infants who were randomized to lactoferrin alone or lactoferrin and probiotics or placebo, the supplementation with lactoferrin alone or in combination with probiotics significantly reduced the incidence of LOS, both fungal and bacterial, in comparison to placebo [109]. Moreover, a recent meta-analysis including 6 randomized controlled trials showed that lactoferrin supplementation to enteral feeds in preterm newborns, alone or in combination with probiotics, reduced the incidence of LOS and NEC stage II or III, although overall mortality was not affected [89]. However, at present, no evidence are available on the possible beneficial effect of lactoferrin administration during neonatal sepsis; therefore, further studies are needed to assess whether lactoferrin could be beneficial not only as a preventive measure but also as an adjunct treatment for neonatal sepsis and also to establish the optimal dosing regimen. Interestingly, in 15 preterm newborns, serum lactoferrin levels were significantly lower in patients with proven sepsis in comparison to those with clinical sepsis and were positively correlated with white blood cell count or absolute neutrophil count, suggesting that the lowest lactoferrin observed in more immature infants is likely related to suboptimal white cell activity and that lactoferrin supplementation could be particularly effective in this population [110].

Vitamin E, which acts primarily as circulating direct antioxidant, has been extensively investigated for the prevention of prematurity-related mortality and morbidity and was proved effective in reducing the incidence of intracranial bleeding and retinopathy of prematurity in the subset of VLBW infants in a meta-analysis including 26 randomized controlled trials [111]. However, vitamin E supplementation was associated with increased risk of sepsis both in the case of intravenous and nonintravenous administration and serum tocopherol levels higher than the cut-off of 3.5 mg/dL were positively associated with increased risk of sepsis [111], leading to the conclusion that routine vitamin E supplementation in preterm newborns cannot be recommended. In a recent randomized open-label study including 65 preterm newborns with mean gestational age of 34 weeks, vitamin E supplementation reduced GPx activity at 30 days in septic newborns in comparison to untreated septic newborn although it also mitigated the reduction of GPx observed in septic patients 60 days after sepsis onset [68]. Moreover, vitamin E supplementation suppressed GR activity in treated septic patients, while increased GPx activity in controls in comparison to untreated controls [68]. The combination of direct scavenging effect of vitamin E with increased GPx activity would result in enhanced H_2_O_2_ removal with a reduction in efficiency of circulating pathogen discharge and therefore would explain the increased risk of sepsis observed in vitamin E-supplemented newborns [68, 111].

Other antioxidant measures with potential efficacy in neonatal sepsis that have been investigated in clinical settings include selenium [112] and zinc supplementation [113], and treatment with ibuprofen [114].

Edaravone (3-methyl-1-phenyl-pyrazolin-5-one), a free radical scavenger introduced in the latest years in experimental settings, exerts multiple antioxidant effects, as hydroxyl radical scavenging, suppression of hydroxyl-dependent lipid peroxidation, and electron donation to ROS [115], leading to beneficial effects in animal models of neonatal hypoxic-ischemic encephalopathy [116]. In a piglet model of neonatal sepsis, edaravone was demonstrated to reduce TH levels 1 hour after CLP and nitrite-nitrate levels at 3 and 6 hours in comparison to septic untreated animals, indicating favorable antioxidant effects [117] (Table 3). These changes were paralleled by clinical improvement of septic animals, as demonstrated by higher cardiac output and mean arterial pressure, lower heart rate, and longer survival time in treated versus untreated animals, suggesting possible beneficial effects of edaravone on sepsis clinical course in the newborns [117]. Furthermore, edaravone delayed TNF-alpha surge in treated animals and also prevented the increase of high mobility group box 1 (HMGB-1), a nuclear transcription factor involved in the systemic inflammatory response [117]. In the same animal models, edaravone was also proved effective in reducing sepsis-related pulmonary hypertension and the ratio between mean pulmonary and systemic arterial pressure was positively related to TNF-alpha levels, suggesting that edaravone may exert suppressive action on TNF-alpha release [118]. In a small cohort of pediatric patients with cerebral infarction, edaravone was recently associated with improved neurological outcome without significant adverse effects [119].

The endothelin system is well known to be involved in sepsis, as endothelin-1 (ET-1) induces activation of NF-*κ*B-mediated proinflammatory pathways and expression of adhesion molecules [120] and ET-1 levels appear increased in septic newborns, particularly in case of pulmonary hypertension [121, 122]. The infusion of endothelin receptor antagonist ETR-P1/fl was proved effective in reducing serum nitrite and nitrate, TNF-alpha, and HMBG-1 in a piglet model of neonatal sepsis and also in reducing pulmonary hypertension and increasing mean arterial pressure and survival time [123] (Table 3). In accordance with this study, in the same animal model, treatment with ETR-P1/fl reduced TH, OSI (calculated as total hydroperoxide/biological antioxidant potentials), and IL-6 at 3 and 6 hours after CLP, indicating attenuation of prooxidant and proinflammatory insult [124]. At present, endothelin receptor antagonists have never been investigated in clinical settings in the newborn.

As dysfunctional mitochondria are the key factor of organ impairment during sepsis [8, 28], mitochondrial-targeted antioxidant treatment has been studied in preclinical models of adult sepsis [125]. In order to achieve antioxidant protection of mitochondria, potential useful strategies include the administration of ROS scavengers, which specifically target the mitochondria and act where needed within the mitochondria, or the induction of endogenous mitochondrial antioxidant system [125]. Particularly, the main studied agents are obtained by conjugation of antioxidant molecules to lipophilic cations that accumulate in the mitochondria, driven by mitochondrial membrane potentials, as MitoQ, containing ubiquinone antioxidant moiety, or by the conjugation of fragment of antibiotic with the stable nitroxide radical TEMPOL, that is able to accept electrons from unstable ROS, to dismute superoxide anion, and to exert catalase-like activity [125]. Other strategies under investigation include the administration of small synthetic peptides (SS peptides) with scavenger activity, which selectively concentrate in the mitochondria, and potentiation of endogenous mitochondrial antioxidant defenses by the administration of N-acetyl-l-cysteine, which accumulates in the mitochondria and increases local glutathione availability or by genetic approaches, as adenoviral transfection with MnSOD [125]. At present, none of these strategies has been studied in models of neonatal sepsis; therefore, mitochondrial-targeted antioxidant treatment could represent a potential future line of research in the field of neonatal sepsis.

## 4. Conclusions

Oxidative stress, along with proinflammatory pathways, was demonstrated to play a major role in neonatal sepsis both in vitro and in vivo. However, few evidences are available at present on the clinical usefulness of adjunct antioxidant treatment in neonatal sepsis.

Some relevant limitations of the studies investigating oxidative stress and antioxidant treatments in neonatal sepsis may partially limit the quality of the available evidence. Most of the studies assessing antioxidant enzyme activity in neonatal sepsis were based on serum measurements that could be influenced by hemolysis processes and in fact provided partially different evidences in comparison to observations based on erythrocyte level measurements [68]. Moreover, data obtained from animal studies on oxidative stress markers or antioxidant treatments in neonatal sepsis may not strictly reflect the clinical conditions of septic newborns, as in experimental settings, the animal models are challenged with high levels of LPS or bacteria, which is often not the case in clinical practice [52, 53, 117, 118, 123, 124]. As regard to clinical observations, not all of the studies reported rigorous inclusion criteria, defining whether both EOS and LOS or only one of the two categories is included; therefore, conclusions derived from these studies could be misleading as redox pathophysiology of EOS and LOS could be partially different. Moreover, while some studies included only culture-proven sepsis, others included both proven and clinical sepsis, and others did not specify this aspect. However, due to the fact that several nonsepsis-related perinatal variables could affect oxidative stress in newborns, only data coming from proven sepsis patients should be considered fully reliable, while those coming from clinical sepsis patients could merely reflect the effect of different conditions activating oxidative stress pathways. Finally, clinical studies investigating the usefulness of antioxidant treatments in neonatal sepsis often lack strict randomization, leading to evidences that, although promising, need to be confirmed by properly designed studies.

Despite several limitations, available evidence suggests that oxidative stress processes are activated during neonatal sepsis, posing the basis for the potential clinical usefulness of antioxidants as an adjunct strategy for the treatment of septic newborns. Antioxidants, in fact, would counterbalance the detrimental prooxidant cycle in newborn sepsis, which, once initiated, proceeds independently from the pathogens themselves and thus is not affected by antibiotic treatment alone. Further studies are needed to identify useful agents and to standardize antioxidant treatment in neonatal sepsis.

## Figures and Tables

**Table 1:** Clinical studies assessing oxidative stress and/or antioxidant defenses in neonatal sepsis.

Subjects	Evaluated markers	Main findings	Ref.
50 newborns: 30 sepsis/20 controls	Serum XO, CPK, SOD, GPx, PO, MDA, uric acid, albumin	Increased XO, CPK, SOD, GPx, MDA, reduced PO, uric acid, albumin in sepsis versus controls	[49]

50 newborns: 30 sepsis/20 controls	Serum TNF-alpha, SOD, GPx	Increased TNF-alpha, SOD, GPx in sepsis versus controls 5-fold increase of TNF-alpha but no differences in SOD, GPx in septic shock versus nonseptic shock	[50]

128 newborns: 44 sepsis/84 controls	Serum MDA, SOD, GPx, CAT, uric acid, albumin	Increased MDA, SOD, GPx, CAT, reduced uric acid, albumin in sepsis versus controls Increased MDA, SOD, GPx, CAT, reduced uric acid, albumin in sepsis-related death versus sepsis survivors	[51]

120 preterm newborns: 20 proven EOS/20 clinical EOS/80 controls Mean GA: 30 wks	Cord blood IL-6, IL-10-TBARS, protein carbonyls	Increased IL-6, IL-10, TBARS, protein carbonyls in proven and clinical sepsis versus controls Increased TBARS, IL-6 in proven versus clinical sepsis TBARS and IL-6 best biomarkers for the diagnosis of sepsis (AUC 0.88) TBARS is the only marker independently associated with EOS	[54]

120 preterm newborns	Serum IL-6, IL-10, TBARS, protein carbonyls	IL-6 and TBARS showed mild to moderate correlation with sepsis severity score No markers predict sepsis-related mortality	[55]

30 term newborns: 20 sepsis/10 controls	Serum MDA + 4-HDA	2-fold increase of MDA + 4-HDA in septic patients versus controls	[63]

52 newborns with LOS: 27 clinical/25 proven LOS Mean GA: 35 wks	Erythrocyte GPx, TrxR, SOD, CAT, selenium, and glutathione; SePP; plasma lipid peroxidation markers	Increased GPx in clinical sepsis versus controls: Reduced TrxR in proven sepsis versus controls: Increased SOD, CAT, lipid peroxidation, reduced selenium, SePP, glutathione in clinical and proven sepsis versus controls	[64]

70 newborns: 35 LOS/35 controls Mean GA: 36 wks	Serum PON-1, TOS, TAS, OSI	Increased TAS/TOS/OSI; reduced PON-1 in sepsis pretreatment versus posttreatment. Increased TAS/TOS/OSI; reduced PON-1 in sepsis pretreatment versus controls	[65]

65 preterm newborns 31 sepsis/34 controls Mean GA: 34 wks	Erythrocyte SOD, CAT, GPx, GR	Increased CAT at 60 days following sepsis diagnosis in sepsis versus controls	[68]

XO: xanthine-oxidase; CPK: creatinine phosphokinase; SOD: superoxide dismutase, GPx: glutathione peroxidase, PO: peroxidase; MDA: malondialdehyde; CAT: catalase; BAP: biological antioxidant potentials; TBARS: thiobarbituric acid reactive species; 4-HAD: 4-hydroxylalkenals; TrxR: thioredoxin reductase; PON-1: paraoxonase-1; GR: glutathione reductase; SePP: selenoprotein P; TOS: total oxidant state; TAS: total antioxidant state; OSI: oxidative stress index.

**Table 2:** Main evidence from clinical studies on melatonin and pentoxifylline treatment in neonatal sepsis.

Enrolled population	Interventional procedure	Outcomes	Ref.
*Melatonin*			

30 newborns: 10 sepsis/10 sepsis and melatonin treatment/10 controls	Melatonin, 20 mg/kg orally within 12 hours of sepsis diagnosis (2 doses, 10 mg/kg each, separated by 1-hour interval)	Reduced MDA + 4-HDA at 1 and 4 hours after treatment in septic treated versus septic untreated infants Reduced WBC count, ANC, CRP 24 hours after treatment in septic treated versus septic untreated infants	[63]

40 newborns: 20 sepsis/20 sepsis and melatonin treatment	Melatonin, 20 mg/kg orally, single dose	Reduced CRP and better clinical improvement at 24 and 72 hours after treatment in treated versus untreated infants	[99]

50 newborns: 25 sepsis/25 sepsis and melatonin treatment	Melatonin, 20 mg/kg orally, single dose	Reduced sepsis score at 24 and 48 hours after treatment in treated versus untreated infants	[100]

*Pentoxifylline*			

120 newborns: 60 LOS/60 LOS and pentoxifylline treatment	Pentoxifylline, 5 mg/kg/h IV for 6 hours for 6 days	Reduced TNF-alpha, vasopressor need, duration of respiratory support, duration of antibiotics, hospital stay, incidence of DIC, and thrombocytopenia in treated versus untreated infants No differences in mortality	[105]

Meta-analysis of 6 randomized or quasi-randomized trials; 416 newborns	Pentoxifylline, continuous IV infusion, different dosing regimens	Reduced all-cause mortality, reduced hospital stay in septic treated versus untreated septic infants Reduced mortality in the subgroup of preterm newborns, proven sepsis, and Gram-negative sepsis in septic treated versus untreated septic infants	[106]

MAD: malondialdehyde; 4-HDA: 4-hydroxylalkenals; WBC: white blood cell; ANC: absolute neutrophil count; CRP: C-reactive protein; DIC: disseminated intravascular coagulopathy; LOS: late-onset sepsis.

**Table 3:** Animal studies of novel antioxidant treatments in neonatal sepsis.

Model	Interventional procedures	Outcomes	Ref.
*Edaravone*			

Piglets	CLP alone or CLP and IV continuous edaravone infusion	Reduced TH at 1 hour after CLP, reduced nitrite-nitrate at 3 and 6 hours, reduced HMGB-1, delayed TNF-alpha surge, increased mean arterial pressure, reduced heart rate, longer survival time in treated versus untreated animals	[117]

Piglets	CLP alone or CLP and IV continuous edaravone infusion	Reduced pulmonary hypertension in treated versus untreated animals Mean pulmonary artery pressure/mean systemic arterial pressure ratio positively related to TNF-alpha levels	[118]

*Endothelin-1 receptor antagonist*			

Piglets	CLP alone or CLP and IV continuous ETR-P1/fl infusion or controls	Reduced nitrite-nitrate, TNF-alpha, HMBG-1, reduced pulmonary hypertension in CLP-treated animals versus CLP alone	[123]

Piglets	CLP alone or CLP and IV continuous ETR-P1/fl infusion or controls	Reduced TH, OSI, IL-6 at 3 and 6 hours post-LP	[124]

CLP: cecal ligation perforation; TH: total hydroperoxide; OSI: oxidative stress index; HMGB-1: high mobility group box 1.
